# Expression, Distribution, and Role of C-Type Lectin Receptors in the Human and Animal Middle Ear and Eustachian Tube: A Review

**DOI:** 10.3390/molecules23040734

**Published:** 2018-03-22

**Authors:** Su Young Jung, Sung Su Kim, Young Il Kim, Hee Yong Chung, Sang Hoon Kim, Seung Geun Yeo

**Affiliations:** 1 Department of Otorhinolaryngology-Head and Neck Surgery, Graduate School, Kyung Hee University, Seoul 02447, Korea; monkiwh35@hanmail.net (S.Y.J.); monkiwh35@naver.com (H.Y.C.); hoon0700@naver.com (S.H.K.); 2 Department of Biochemistry and Molecular Biology, Medical Science and Engineering Research Center for Bioreaction to Reactive Oxygen Species, BK-21, School of Medicine, Kyung Hee University, Seoul 02447, Korea; sgskim@khu.ac.kr; 3 Medical Science Research Institute, Kyung Hee University Medical Center, Seoul 02447, Korea; ewlabkim@khmc.or.kr

**Keywords:** lectin, glycans, c-type lectin receptor, human middle ear, otitis media

## Abstract

Otitis media (OM) is a group of inflammatory diseases of the middle ear (ME), regardless of cause or pathological mechanism. Among the molecular biological studies assessing the pathology of OM are investigations into the expression of C-type lectin receptors (CLR) in the ME and Eustachian tube (ET). To date, nine studies have evaluated CLR expression in the ME and ET. The expression of individual CLRs in mammalian ME and ET varies by species and model of OM. Assessments have shown that the patterns of CLR expression in the ME and ET vary; that CLR expression may vary by type of OM; and that the distribution and levels of expression of CLRs may depend on the presence or absence of inflammation, with variations even within the same species and same tissue. Infection of the ME and ET with various pathogens is a common cause of all types of OM, with host responses to pathogens mediated initially by the innate immune system. CLRs are important factors in the innate immune system because they act as both adhesion molecules and as pathogen recognition receptors. The expression of CLRs in OM tissues suggests that CLRs are associated with the pathogenesis of various types of OM.

## 1. Introduction

**Figure 1:**
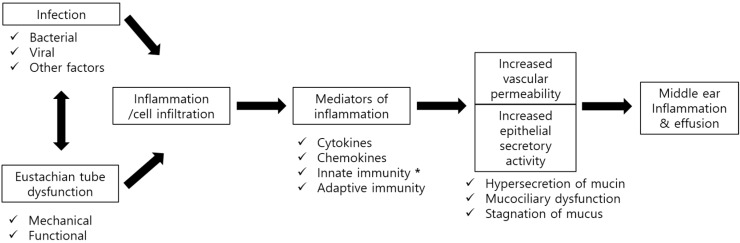
General pathogenesis of otitis media.

Otitis media (OM) is a group of inflammatory diseases of the middle ear (ME), regardless of cause or pathological mechanism (Figure 1) [1]. OM can be categorized by cause, clinical course, and pathologic mechanism. In clinical practice, OM can be classified as acute otitis media (AOM), otitis media with effusion (OME), chronic suppurative otitis media (COM), or otitis media with cholesteatoma (Chole OM) [2]. Various factors play a role in the pathogenesis of each type of OM. Numerous studies designed to elucidate the pathogenesis of OM in animals, as well as assessments of the histopathology of the temporal bone in humans, have suggested that the histopathological, biochemical, and immunological findings in different types of OM are closely correlated with each other, leading to transitions among the different types of OM [3,4,5,6]. The most important contributing factors are Eustachian tube (ET) dysfunction and microbial infection [7], which induce ME inflammation. Subsequently, the pattern and progression of OM vary depending on each individual’s conditions, immune responses, and biochemical factors, all of which are thought to be related to the recurrence and chronic character of OM.

Among the molecular biological studies assessing the pathology of OM are investigations of the expression of lectins in the ME cavity and ET. Lectins are carbohydrate-binding proteins that are highly specific for sugar moieties. Lectins recognize binding molecules at both the cellular and molecular levels and play numerous roles in biological recognition phenomena involving cells, carbohydrates, and proteins [8,9]. Lectins also mediate the attachment and binding of bacteria, viruses, and fungi to their targets. Lectins have many biological functions in animals, from regulating cell adhesion to glycoprotein synthesis and the control of protein levels in the blood. Lectins may also bind soluble extracellular and intercellular glycoproteins. 

Lectins also play important roles in the immune system. Glycoproteins, including those containing pathogen-associated molecular patterns (PAMPs), are found on the surfaces of many pathogens. Some lectins act as pattern-recognition receptors (PRRs), binding to these PAMPs on invading microorganisms and malignant, apoptotic, or dead host cells. This binding can lead to the induction of an immune response against the invading pathogen. However, the relationship between lectins and various pathogens is complex. In addition to immune evasion, glycosylation is essential for protein expression and assembly and for entry into cells during inflammation [10], and many pathogens have evolved mechanisms to exploit lectins to enhance infection. The distribution of various lectins in the ear may be closely correlated with both normal physiology and the pathophysiology of OM, especially regarding the host immune system in the ET and ME. This review analyzes the characteristics of lectins that play important roles in host responses against pathogens in the ET and ME.

## 2. Classification of Lectin Families

Complex oligosaccharide structures are present at cell surfaces, incorporated into extracellular matrices, and attached to secreted glycoproteins. These oligosaccharides can serve structural roles, mediate the movement of glycoconjugates to the cell surface, or act as markers that mediate cell–cell and cell–matrix recognition events. The nonstructural roles of sugars generally require the participation of sugar-binding lectins, in which sugar-binding activity can usually be ascribed to a single protein module within the lectin polypeptide [11]. Such a module is referred to as a carbohydrate-recognition domain (CRD). Most lectins are members of families with defined CRDs, which apparently evolved from shared ancestral genes, often retaining specific primary amino acid sequences or three-dimensional structures. The general characteristics of lectins are summarized in Table 1 [12].

CRDs in vertebrate lectins fall into a number of structurally distinct families. Of the eight well-established groups of lectins, four are predominantly intracellular and four generally function outside cells. The intracellular lectins—the calnexin family, and M-type, L-type, and P-type—are located in luminal compartments of the secretory pathway and function in the trafficking, sorting, and targeting of maturing glycoproteins. The extracellular lectins—C-type, R-type, siglecs, and galectins—are secreted into the extracellular matrix or body fluids, or localize to the plasma membrane, and mediate a range of functions including cell adhesion, cell signaling, glycoprotein clearance, and pathogen recognition. Recent findings indicate that there are additional groups of animal lectins—F-box lectins, ficolins, chitinase-like lectins, F-type lectins, and intelectins—some of which have roles complementary to those of the well-established lectin families [11]. Table 2 shows a brief summary of these various lectin families [13].

## 3. Innate Immunity and C-Type Lectin Receptors (CLRs)

### 3.1. Innate Immunity and Pattern Recognition Receptors (PRRs)

Host responses against pathogens are mediated initially by the innate immune system and later by the adaptive immune system, involving various cytokines, chemokines, and co-stimulatory signals expressed by antigen-presenting cells (APCs) [14,15]. T cells and B cells are responsible for adaptive immune responses, which are characterized by antigenic specificity and memory against specific antigens. Although the innate immune response does not involve antigenic specificity or memory against specific antigens, it is characterized by a rapid response against external stimuli. Epithelial cells express various PRRs, allowing them to respond to stimuli such as antigen exposure or infection. These cells secrete various types of cytokines and chemokines, which recruit immune system cells to infected areas and generate signals to activate defense mechanisms [16]. PAMPs are molecules associated with specific groups of pathogens, and the characteristic structures of PAMPs are recognized by cells of the innate immune system. Innate immunity is an evolutionarily conserved system that provides the body’s first line of defense against invading viral and microbial pathogens and helps maintain homeostasis by regulating endogenous processes such as inflammation and cell death. Many host cells express receptors, called PRRs, that mediate cellular responses against pathogens by recognizing molecules, such as PAMPs, associated with pathogens or extracellular damage [17]. Among these PRRs are Toll-like receptors (TLRs), NOD-like receptors (NLRs), RIG-I-like receptors (RLRs), and CLRs. Aberrant expression and excessive immune responses of these PRRs induce inflammatory diseases [18].

### 3.2. C-Type Lectin Receptors (CLRs)

CLRs comprise a large family of receptors that bind to carbohydrates in a calcium-dependent manner. The lectin activity of these receptors is mediated by conserved CRDs. Compared with other PRRs, CLRs are especially important for APCs such as dendritic cells (DCs), which recognize, take up, and process antigens, eventually presenting them to effector cells. Relative to other PRRs, the number of CLRs is greater, and their morphology and types are more varied. These CLRs are expressed on the surfaces of APCs, allowing recognition of various glycosylation patterns in many different antigens, followed by the binding and uptake of these antigens. Therefore, CLRs not only recognize and take up various antigens for their removal, but also transmit signals within cells, assisting macrophages and dendritic cells in the effective induction of innate immunity [19].

Based on their molecular structures, there are two forms of CLRs—soluble and transmembrane CLRs. Soluble CLRs form a subclass of lectins with a characteristic composition and function due to their solubility in plasma and their presence on mucosal surfaces. Recognition of microbes by soluble CLRs may result in the removal of these microbes by opsonization, complement activation, initiation of phagocytosis, and/or inhibition of microbial growth. Soluble CLRs are also involved in modulating adaptive immune responses by forming connections between APCs and the carbohydrates present on microbes. Structurally, soluble CLRs lack signaling motifs. Rather, immune responses are triggered after opsonization by phagocytic cells. Soluble C-type lectins belonging to the family of collagen-containing C-type lectins (collectins) include MBL, lung surfactant proteins A (SP-A), SP-D, and collectin kidney 1 (CL-K1) [20].

Transmembrane CLRs are generally categorized as type I or type II receptors based on their molecular structure. The receptors of both groups express one or more extracellular CRDs, a stalk region, a TM region, and an intracellular part with or without a signaling motif. The N-termini of type I CLRs are located on the exterior of cells, whereas the N-termini of type II CLRs are located within the cytoplasm. In addition, type I CLRs express various CRDs on their extracellular C-type lectin domains (CTLD), whereas type II CLRs only possess single CRDs at their C-terminus [21]. Type I transmembrane CLRs include the family of mannose receptors, such as DEC-205 (also known as CD205 and LY75) and the mannose receptor (MR, also known as CD206), both of which are important in antigen uptake. Type II transmembrane CLRs typically carry a single CRD domain and include DC-associated C-type lectin 1 (Dectin-1, also known as CLCE7A), Dectin-2 (also known as CLEC6A), macrophage-inducible C-type lectin (Mincle, also known as CLEC4A), the dendritic-cell-specific ICAM3-grabbing nonintegrin (DC-SIGN, also known as CD209), and DC NK lectin group receptor-1 (DNGR-1, also known as CLEC9A). These receptors are involved in the recognition of various pathogens and the modulation of the innate immune response. CLRs are expressed by most cell types, including macrophages and DCs, which phagocytose various glycoproteins and microbes, both for clearance and antigen presentation to T lymphocytes. CLRs of DCs show interactions among mannose, fucose, and glucan carbohydrates, all of which include pathogens infectious to humans. Mannose is present in viruses, fungi, and mycobacteria, whereas fucose is expressed by specific bacteria and parasites. Glucan structures are expressed by fungi and mycobacteria. CLRs allow pathogens to enter DCs, both to degrade these pathogens and to generate antigens [22].

A comparison of the structures of transmembrane and soluble CLRs showed that transmembrane CLRs form a high-density receptor pattern on the host cell membrane, enhancing strong interactions on small surfaces. In contrast, soluble CLRs tend to form more widely stretched multimeric conformations that surround microbial agents.

## 4. Types and Functions of CLRs Expressed in the Middle Ear and Eustachian Tube

### 4.1. Expression of CLRs in the Middle Ear and Eustachian Tube 

To date, nine studies have evaluated the expression of CLRs in the ME and ET [23,24,25,26,27,28,29,30,31] (Table 3). These include one study in mouse, one in rat, and seven in human patients. Although the expression of CLRs varied by species and model, various types of CLRs were found to be expressed in mammalian ME and ET, suggesting that lectins may play various roles in the pathophysiology of OM.

### 4.2. Pathophysiologic Roles of CLRs Expressed in the Middle Ear and Eustachian Tubes

#### 4.2.1. Mannose-Binding Lectin (MBL, Also Called Mannan-Binding Lectin)

MBL is a soluble CLR belonging to the class of collectins. Functionally, MBL is involved in pattern recognition, as part of the first line of defense in pre-immune hosts. MBL recognizes carbohydrate patterns on the surfaces of a large number of pathogenic micro-organisms, including bacteria, viruses, protozoa, and fungi. Binding of MBL to a microorganism activates the lectin pathway of the complement system. In addition, MBL binds to senescent and apoptotic cells, enhancing the engulfment by phagocytes of whole, intact apoptotic cells, as well as of cell debris [32,33,34]. MBL also interacts with carbohydrates on glycoprotein (gp) 120 of HIV-1 and may inhibit DC-SIGN-mediated uptake and spread of HIV [35].

Three studies to date have analyzed MBL expression in OM—two targeting middle ear effusion (MEE) in patients with OME [29], and one targeting the cholesteatoma matrix of patients undergoing surgery for Chole OM [23]. In the former study, 73 patients with childhood OME were divided into three groups based on their MEE characteristics: an otitis prone group, a recurrent acute OM group, and a secretory OM group. Double enzyme immune-assays (EIA) of MEE collected from these patients showed no differences in the levels of MBL expression among these three groups [29]. Similarly, a statistically significant positive correlation was observed between mannan-binding protein (MBP) concentrations in nasopharyngeal secretions and MEE (*n* = 42; r = +0.47, *p* = 0.02), suggesting that MBL takes part in the pathophysiology of OME by affecting local mucosal defense mechanisms. In MEE, MBL was reported to bind to *Alloiococcus otitidis* in a calcium-dependent manner, with immunoblotting analysis confirming the presence of MBL in the MEE of patients with childhood OME [30]. These findings suggested that, by enhancing the phagocytosis of macrophages, MBL provides protection against OM caused by *A. otitidis* infection through the innate immune system [30]. Because MBL activates the lectin pathway of the complement system by binding to targets such as mannose on pathogens, such as yeasts, viruses, bacteria, and parasites, and enhances phagocytosis, it is likely important for the pathophysiology of OM, including the induction of innate immunity.

#### 4.2.2. Surfactant Proteins (SP)-A and -D

Lung surfactant proteins (SP)-A and -D are located in the surfactant present at the luminal surfaces of pulmonary epithelial cells. SP-A binds to mannose residues, as well as binding to fucose with higher affinity [36]. SP-A was shown to be involved in regulating surfactant phospholipid pools in alveolar cavities by CRD-dependent phospholipid binding [37]. In addition, SP-A can interact with lipids via its collagen-like region. The role of SP-A in immune regulation remains unclear. SP-A forms bouquet-like octadecamers via the formation of intermolecular disulphide bonds at the N-terminus, followed by further self-association via reversible formation of calcium-dependent supraquaternary structures. These complex supraquaternary structures bind to phospholipids and other protein structures and enhance immunological functions and biophysical effects, including the reduction of surface tension at the air–liquid interface by binding dipalmitoylphosphatidylcholine (DPPC) [38]. 

The lectin receptor SP-D binds to maltose, mannose, and glucose residues. In addition, SP-D CRDs recognize lipopolysaccharides of Gram-negative bacteria. For example, in mice, SP-D was shown to rapidly opsonize nonencapsulated *Klebsiella pneumonia* expressing mannose-containing O-antigens, followed by macrophage production of IL-1β and IL-6 and eradication of the bacterium [39]. In addition, the CRD of SP-D can bind to high-mannose oligosaccharides present on the hemagglutinin (HA) coat of most strains of influenza virus [40]. In addition to binding to carbohydrates, SP-D was shown to bind phosphatidylinositol and glucosylceramide [41]. 

Immunoblotting analysis of MEE extracted from patients with OME showed that SP-A adheres to pathogens in a calcium-dependent manner [30]. Using a mouse model of OME, both SP-A and SP-D were found to be expressed in normal murine ET and ME tissues, with SP-A being especially up-regulated by stimulation with lipopolysaccharide (LPS) [24]. This up-regulation of SP-A was accompanied by increased concentrations of proinflammatory cytokines, including tumor necrosis factor-α (TNF-α), interleukin (IL)-1 β, and IL-6. These findings indicate that SP-A and SP-D, both of which are soluble CLRs, play a key role, along with certain proinflammatory cytokines, in pathogen infection during the pathogenesis of OM.

#### 4.2.3. Type I Transmembrane CLRs

Mannose receptor (MR, also known as CD206) and DEC-205 (also known as CD205) are type I transmembrane CLRs containing several CRDs or CRD-like domains. MR is primarily present on the surfaces of macrophages and immature DCs, but is also expressed on the surfaces of skin cells, such as human dermal fibroblasts and keratinocytes [42,43]. The functions of MR can be subdivided into five different categories: (1) phagocytosis of pathogens, (2) antigen presentation, (3) intracellular signaling, (4) resolution of inflammation, and (5) clearance of glycoprotein hormones. A number of pathogenic microorganisms, including *C. albicans*, display glycans on their surfaces with terminal mannose residues that are recognized by the C-type CRDs of the mannose receptor, thereby acting as a marker of non-self. Upon recognition, the receptor internalizes the bound pathogen and transports it to lysosomes for degradation via the phagocytic pathway. In this way, the mannose receptor acts as a PRR [44]. Surprisingly, MR knockout mice did not show increased susceptibility to infection, suggesting that this receptor is not essential for phagocytosis. However, it may still be involved in phagocytosis, as other mechanisms may compensate for the absence of MR. For example, infection of knockout mice with *P. carinii* increased the recruitment of macrophages to the site of infection. Furthermore, other receptors present on the surface of phagocytic cells, such as DC-SIGN, SIGNR1, and Endo180, exhibit ligand binding ability similar to that of MR, suggesting that these proteins can compensate for the absence of MR and induce phagocytosis [45]. In addition, MR may also play a role in antigen uptake and presentation by immature DCs during adaptive immune responses. Mature DCs and macrophages use MR for antigen presentation in a different way. The cleaved, soluble receptor binds to circulating antigens via its cysteine-rich domain, and directs these antigens to effector cells in lymphoid organs, thus activating the adaptive immune system [46].

Two studies to date have assessed the relationship between OM and MR; one analyzed MR expression in MEE of patients with OME [26] and the other in the cholesteatoma matrix of patients with Chole OM [23]. MR was expressed in MEE of OME patients. Moreover, although the level of MR expression was not influenced by the characteristics of OME exudates or the presence of bacteria, MR expression was higher in patients with than without accompanying diseases, such as sinusitis, adenoid vegetation or adenoiditis, and allergic rhinitis [26]. MR expression was also higher in the cholesteatoma matrix collected during surgery on patients with Chole OM than in normal skin tissue [23], suggesting that a high level of MR expression in cholesteatoma tissue is likely important in inducing innate immune responses against pathogens, as well as playing an important role in infection and inflammation due to cholesteatoma. Moreover, the higher levels of MR expression in cholesteatoma than in normal tissue indicate that MR is involved in the progression of cholesteatoma from recurrent infection and chronic inflammation.

DEC-205 is predominantly expressed by the thymic cortical epithelium and by DCs in mice, humans, and other species [47,48]. DEC-205 plays an important role in antigen uptake for presentation and cross-presentation to T cells. Moreover, antigen uptake via DEC-205 in the steady-state results in immune tolerance, suggesting that DEC-205 plays an important role in inducing tolerance of CD4 and CD8 T cells to self-antigens, both at the periphery and in the thymus [49]. 

Three studies of the relationship between OM and DEC-205 confirmed that DEC-205 was expressed in the middle ear tissues of patients with both Chole OM [23,25] and OME [26]. The levels of DEC-205 expression were unrelated to exudate characteristics, the presence or absence of bacteria, or erosion of ossicles. Because DEC-205 is a type of CLR that commonly acts on innate immunity, specifically during early stages of host defense, various factors accompanying lesions of the middle ear likely did not affect DEC-205 expression during chronic inflammation.

#### 4.2.4. Type II Transmembrane CLRs Associated with OM

Type II transmembrane CLRs typically carry a single CRD domain and include Dectin-1, Dectin-2, macrophage-inducible C-type lectin (Mincle), the DC-specific ICAM3-grabbing nonintegrin (DC-SIGN), and DC NK lectin group receptor-1 (DNGR-1). These receptors are involved in recognition of fungi and modulation of the innate immune response [50]. 

Dectin-1 plays an important role in antifungal innate immunity. Dectin-1 is a specific receptor for β-glucans [51]—glucose polymers found in the cell walls of fungi, including the yeasts *Saccharomyces cerevisiae* and *Candida albicans*. Dectin-1 signaling has been shown to synergize with TLR2 signaling in enhancing the responses triggered by each receptor alone [52,53]. 

Dectin-2 is also important in antifungal innate immunity. Dectin-2 binds high-mannose-type carbohydrates and was shown to be the functional receptor for α-mannans. Moreover, Dectin-2 has been implicated in antibacterial immunity and allergy [54]. Like Dectin-1, Dectin-2 belongs to the selective group of CLRs that link pathogen recognition to adaptive immunity. Dectin-2 was shown to be the predominant receptor in response to fungal infection and the induction of Th17 immunity. Similar to Dectin-1, activation of Dectin-2 triggers the generation of reactive oxygen species (ROS) and potassium efflux, leading to NLRP3 inflammasome activation and processing of pro-IL-1β [55].

Mincle is a member of the Dectin-2 family that recognizes various exogenous and endogenous stimuli, such as mycobacteria, certain fungi, and necrotic cells [56]. Exogenous ligands for Mincle include fungal α-mannose and the mycobacterial glycolipid, trehalose-6′6′-dimycolate (TDM), also known as cord factor, the immunostimulatory component of *Mycobacterium tuberculosis* [57]. Mincle interacts with the Fc receptor common γ-chain (FcRγ), which triggers intracellular signaling through Syk, leading to CARD9-dependent NF-κB activation. Syk also induces the mobilization of intracellular calcium and the activation of the calcineurin-NFAT pathway.

DC-SIGN is of interest due to its involvement in the recognition of several viruses, including HIV-1, HCV, dengue virus, CMV, and Ebola virus, as well as other microbes of the genera *Leishmania* and *Candida*. This type II transmembrane protein has a single C-type lectin domain and is expressed on immature monocyte-derived DCs. DC-SIGN modulates TLR signaling at the level of the transcription factor NF-κB, although prior TLR activation of NF-κB is required. Pathogens have been shown to trigger DC-SIGN on human DCs, activating the serine and threonine kinase Raf-1, which subsequently leads to the acetylation of the NF-κB subunit p65. Acetylation of p65 both prolongs and increases IL-10 transcription, enhancing anti-inflammatory cytokine responses [58]. 

DNGR-1 (CLEC9A) is particularly interesting because of its restricted pattern of expression in DCs, a pattern that may be exploited for the treatment of cancer. DNGR-1 was recently shown to bind to damaged or dead cells via exposed actin filaments [59]. Because no microbial ligand has yet been identified, DNGR-1 is considered a DAMP receptor.

Trem-1 is up-regulated by bacterial LPS and mediates the activation of neutrophils and monocytes. In addition, Trem-1 plays an important role in immune responses by inducing the secretion of inflammatory chemokines and cytokines and the release of myeloperoxidase, as well as by up-regulating adhesion molecules [60].

Tim-3 is highly expressed in differentiated Th1 cells and is present at lower levels in monocytes, natural killer (NK) cells, and NK T cells. Recently, Tim-3 was shown to regulate innate immune responses and to be associated with allergy and autoimmune diseases [61]. 

Bcl-10 is an intracellular NF-kB activator with a caspase recruitment domain (CARD) that can form homo-oligomers through CARD–CARD interactions under suitable conditions. LPS signaling was found to be considerably reduced in the marginal zone and follicular B cells of Bcl-10 knockout mice, enabling these mice to be more easily infected with *Streptococcus pneumoniae*. In addition, these mice were unable to survive infection, due to the failure of their immune systems to eliminate bacteria. Bcl-10 is also crucial in innate resistance to bacterial infection and LPS signaling [62].

Three studies to date have assessed the expression of type II transmembrane CLRs in patients with Chole OM and OME. Using RT-PCR, these studies confirmed that mRNAs encoding type II transmembrane CLRs were expressed in all types of OM [23,26,27]. An assessment of MEE from patients with OME showed that the levels of expression of type II transmembrane CLR mRNAs were higher in patients with than without accompanying diseases, such as allergic rhinitis, sinusitis, and adenoid vegetation [26]. Because all of these are comorbid diseases in patients with OM, these CLRs function as local sources of mucosal infection and activate host defense mechanisms within the ME or ET. Two studies have assessed the levels of mRNA encoding type II transmembrane CLRs in cholesteatoma matrix obtained during surgery from patients with Chole OM. Both studies found that the level of expression did not depend on the presence of otorrhea or bacteria. However, the levels of the CLR mRNAs were higher in the cholesteatoma matrix than in skin from the same patient [23,25]. These findings suggested that CLRs are involved in the pathophysiology of Chole OM and play specific roles in infection and inflammation during innate immune responses, thereby affecting cholesteatoma formation.

#### 4.2.5. Selectin

Selectins are especially important in leukocytes rolling along vessel walls prior to their firm attachment and extravasation during inflammation [63,64]. Selectins and other adhesion molecules cooperate with a variety of cell-associated and soluble mediators of inflammation, such as platelet-activating factor and IL-8, to modulate inflammatory reactions [65]. L-selectin appears to participate in the adhesion of neutrophils, monocytes, and lymphocytes to activated endothelium by recognition of vascular mucin-like receptors [66]. In addition, L-selectin was shown to mediate rolling of polymorphonuclear leukocytes (PMNs) along the walls of the microvasculature, a phenomenon that may be the first step in the accumulation of leukocytes at sites of inflammation. L-selectin is constitutively expressed on the surface of leukocytes and released by shedding after stimulation [67].

Three studies to date have investigated the relationship between OM and selectins. A study assessing the expression of L-selectin in MEE samples of patients with OME found that the concentration of soluble L-selectin in MEE from children with all types of OM was higher than that in normal serum. This was especially evident in acute purulent effusion samples, in which the expression of soluble L-selectin was three times higher than that in other types of effusion samples, suggesting that soluble L-selectin plays a key role in the pathophysiology of AOM [27]. Soluble L-selectin was shown to beneficially block leukocyte extravasation during pathologic inflammatory responses [68]. Following leukocyte activation, L-selectin is shed by proteolytic cleavage from the surfaces of leukocytes [67]. This soluble L-selectin retains bioactivity and, at high concentrations, can inhibit leukocyte adhesion to endothelium by competitive binding to mucin-like receptors [69,70,71]. The shedding of high amounts of L-selectin during AOM may interfere with further leukocyte rolling and extravasation. 

Another study assessed the expression of granule membrane protein-140 (GMP-140), a member of the selectin family, in MEE. Compared with effusion fluid from patients with other types of OMs and serum, effusion fluid from patients with acute purulent OM had the highest level of GMP-140 [28]. GMP-140 can play a fundamental role in both limiting and mediating the inflammatory responses of neutrophils [72]. Using a rat model of OME, the fluorescence intensity of L-selectin was found to be lower in PMNs from MEE than in isolated PMNs from ME tissue [28]. The percentage of L-selectin (+) cells must have been lower in PMNs from MEE than from peripheral blood, despite the expression of L-selectin in peripheral PMNs decreasing after incubation with MEE. PMNs activated by chemoattractants shed L-selectin from their surfaces [73]. This result also indicated that PMNs were continuously activated by chemoattractants in MEE, which would explain the minimal expression of L-selectin on infiltrated cells in MEE.

#### 4.2.6. Additional Experiments

Six studies to date have investigated OM-associated changes in glycosylation pattern or cell surface carbohydrates using lectins in ME and ET [74,75,76,77,78,79]. These studies utilized 5–7 types of fluorescein-labeled lectin. Three of these studies assessed lectin-induced changes in cell surface carbohydrates in ME or ET mucous membrane tissue, one investigated changes in glycosylation pattern, one assessed changes in glycoconjugates, and one analyzed the binding level of *N*-acetylgalactosamine (GalNac) residues. Lectins are identified with distinct carbohydrates on glycoconjugates that mediate cell–cell recognition, adherence, and interaction [80]. The mode of interaction between lectins and carbohydrate receptors is similar to that between ligands and receptors, in that there is a spatial requirement for lectin (ligand) to bind to carbohydrate (receptor). Alterations in the carbohydrates of glycoconjugates have been associated with many diseases, especially inflammatory diseases [81,82]. Therefore, studying changes of carbohydrates in ME or ET using lectins is vital in understanding the pathophysiology of OM. In normal ET, the levels of GalNac, Galβ(1-3)GalNAc, L-fucose, and NeuNAcα(2-3/2-6)GalNAc in glycoconjugates were high, whereas the levels of a-mannose and sialic acid were low. In contrast, glycoconjugate expression by cilia and goblet cells was low in ET tissues from patients with SOM and mucoid OM [75], which may reflect an OM-associated disorder of carbohydrate metabolism in the cilia of the ET. Characterizing the pattern of carbohydrate receptor expression in normal ET epithelium is a first but important step toward understanding the biological structure of adhesive molecules on cell surfaces and in goblet cells. This may provide a molecular basis for understanding the pathogenesis of diseases such as OM, as these molecules are involved in host–pathogen recognition and adherence. Also, as OM is frequently triggered by bacterial infection, it is critical to determine mechanisms by which the host detects bacteria in mucous membranes of the ME and their associations with host defense mechanisms.

Bacterial adherence is the mechanism by which bacteria attach to mucosal surfaces. This involves the interaction of bacterial adhesins, which are binding molecules expressed on bacterial cell surfaces, often in the form of fimbriae or pili, with receptors on host cell surfaces [83]. These host cell surface receptors are mainly comprised of glycolipids and glycoproteins in the cell membrane. Cell surface carbohydrates in the ET and ME of chinchillas were found to depend on the length of exposure to *Streptococcus pneumoniae* (Spn), a major pathogen of OM [76,77]. Thus, host immune responses are regulated by the time of exposure to pathogens that cause OM, such as Spn, with lectins being involved in this regulation.

## 5. Conclusions

This review has summarized current knowledge about the distribution and function of CLRs in ME and ET, and their associations with mechanisms that may influence the pathophysiology of OM. This review showed that (1) various types of CLRs are expressed in the ME and ET; (2) CLR expression may vary by type of OM; and (3) the distribution and levels of expression of CLRs may depend on the presence or absence of inflammation, with variations even within the same species and same tissue.

Bacterial infection in the ME and ET is a common pathological mechanism for all types of OM, causing tissue edema and inducing inflammation—processes that may involve various CLRs. The expression patterns of several CLRs were found to be altered in response to inflammatory stimuli, suggesting that CLRs may have immunological functions in OM. Additional studies are required to determine the expression and function of CLRs in the physiological and pathological states of the ME and ET, as well as to identify the factors that interact with CLRs and to determine how CLRs are modulated by these factors.

## Figures and Tables

**Table 1:** General characteristics of lectins.

Characteristics	Explanation
Defining arrangement of amino acid residues involved in binding	Often typical for each group
Location of cognate residues within glycans	Typically in sequences at outer ends of glycan chains
Shared evolutionary origins	Yes (within each group)
Shared structural features	Yes (within each group)
Single-site binding affinity	Often low; high avidity generated by multivalency
Specificity for glycans recognized	Stereospecificity high for specific glycan structures
Subgroups	C-type lectins, galectins, Galectins (S-type lectin), P-type lectins (M6P receptors), I-type lectins, L-type lectins, R-type lectins, etc.
Type of glycans recognized	N-glycans, O-glycans, glycosphingo-lipids (a few also recognize sulfated glycosaminoglycans)
Types of glycans recognized within each group	May be similar (e.g., galectins) or different (e.g., C-type lectins)
Valency of binding sites	Multivalency common (either within native structures or by clustering)

**Table 2:** Summary of lectin families.

Lectin Family	Typical Saccharide Ligands	Subcellular Location	Examples of Functions
Calnexin	Glc1Man9	ER	Protein sorting in the endoplasmic reticulum.
Chitinase-like lectins	Chito-oligosaccharides	Extracellular	Collagen metabolism (YKL-40).
C-type lectins	Various	Cell membrane, extracellular	Cell adhesion (selectins), glycoprotein clearance, innate immunity (collectins).
F-box lectins	GlcNAc2	Cytoplasm	Degradation of misfolded glycoproteins.
Ficolins	GlcNAc, GalNAc	Cell membrane, extracellular	Innate immunity.
F-type lectins	Fuc-terminating oligosaccharides	Extracellular	Innate immunity.
Galectins	β-Galactosides	Cytoplasm, extracellular	Glycan crosslinking in the extracellular matrix.
I-type lectins (siglecs)	Sialic acid	Cell membrane	Cell adhesion.
Intelectins	Gal, galactofuranose, pentoses	Extracellular/cell membrane	Innate immunity; fertilization and embryogenesis.
L-type lectins	Various	ER, ERGIC, Golgi	Protein sorting in the ER.
M-type lectins	Man8	ER	ER-associated degradation of glycoproteins.
P-type lectins	Mannose 6-phosphate, others	Secretory pathway	Protein sorting post-Golgi, glycoprotein trafficking, ER-associated degradation of glycoproteins, enzyme targeting.
R-type lectins	Various	Golgi, Cell membrane	Enzyme targeting, glycoprotein hormone turnover.

ER, endoplasmic reticulum; YKL-40, Chitinase-2-like protein 1; ERGIC, endoplasmic reticulum–Golgi intermediate compartment; GlcNAc, *N*-acetylglucosamine; GalNac, *N*-acetylgalactosamine.

**Table 3:** Studies assessing the expression of C-type lectin receptors (CLRs) in the middle ear and Eustachian tube.

Authors and References	Species	Experimental Conditions	Type of CLRs	Detection Methods
Kim et al. [23]	Human	Chole OM	MBL, CD206, DEC-205, DC-SIGN, Langerin, MGL, CLEC5A, Dectin-2, BDCA2, Mincle, DCIR, Dectin-1, MICL, CLEC2, DNGR1, CLEC12B	Real-time PCR
Li et al. [24]	Mouse	OME	SP-A, SP-D	Reverse transcription PCR, Real-time PCR
Kim et al. [25]	Human	Chole OM	DEC205, Bcl-10, Tim-3, Trem-1	Real-time PCR
Lee et al. [26]	Human	OME	Dectin-1, MR1, MR2, DC-SIGN, Syk, Card-9, Bcl-10, Malt-1, Src, DEC205, Galectin-1, Tim-3, Trem-1, DAP-12	Real-time PCR
Pospiech et al. [27]	Human	OME	Soluble L-selectin	ELISA, Bradford assay
Himi et al. [28]	Human	OME	Soluble ICAM-1, soluble GMP-140	ELISA
Garred et al. [29]	Human	OME	MBL	EIA
Konishi et al. [30]	Human	OME	MBL, SP-A	Immunoblotting analysis
Kamimura et al. [31]	Rat	OME	L-selectin	Flow cytometry

Chole OM, otitis media with cholesteatoma; MBL, mannose-binding lectin; Dectin-1, dendritic-cell-associated C-type lectin-1; MR1, mannose receptor 1; MR2, mannose receptor 2; DC-SIGN, dendritic-cell-specific ICAM3-grabbing non-integrin; DEC-205, dendritic and epithelial cell-205; Tim-3, T-cell immunoglobulin mucin-3; MGL, macrophage galactose lectin; CLEC5A, C-type lectin domain family 5 member A; BDCA-2, blood dendritic cell antigen 2; DCIR, dendritic cell immunoreceptor; MICL, myeloid inhibitory C type-like lectin; CLEC2, C-type lectin-like receptor 2; DNGR1, DC NK lectin group receptor-1; CLEC12B, C-type lectin domain family 12, member B; PCR, polymerase chain reaction; OME, otitis media with effusion; SP-A, surfactant protein-A; SP-D, surfactant protein-D; Bcl-10, B cell lymphoma 10; Trem-1, triggering receptor expressed on myeloid cells-1; Syk, spleen tyrosine kinase; Card-9, caspase recruitment domain family member 9; Malt-1, mucosa-associated lymphoid tissue lymphoma translocation gene 1; Src, steroid receptor co-activator; DAP-12, DNAX-activating protein of 12 kDa; ELISA, enzyme-linked immunosorbent assay; ICAM-1, intercellular adhesion molecule 1; GMP-140, granule membrane protein-140; EIA, enzyme immuno-assay.
